# New records and checklist of Chilocorini (Coleoptera: Coccinellidae) from China

**DOI:** 10.3897/BDJ.8.e51092

**Published:** 2020-06-24

**Authors:** Wenjing Li, Bingxu Chen, Lizhi Huo, Xiaosheng Chen, Xingmin Wang

**Affiliations:** 1 Guangdong Provincial Key Laboratory of High Technology for Plant Protection, Plant Protection Research Institute, Guangdong Academy of Agricultural Sciences, Guangzhou, China Guangdong Provincial Key Laboratory of High Technology for Plant Protection, Plant Protection Research Institute, Guangdong Academy of Agricultural Sciences Guangzhou China; 2 Key Laboratory of Bio-Pesticide Innovation and Application, Engineering Technology Research Center of Agricultural Pest Biocontrol, Guangdong Province; Engineering Research Center of Biological Control, Ministry of Education & Guangdong Province, South China Agricultural University, Guangzhou, China Key Laboratory of Bio-Pesticide Innovation and Application, Engineering Technology Research Center of Agricultural Pest Biocontrol, Guangdong Province; Engineering Research Center of Biological Control, Ministry of Education & Guangdong Province, South China Agricultural University Guangzhou China

**Keywords:** Coleoptera, Coccinellidae, Chilocorini, new record, checklist, China

## Abstract

**Background:**

China is one of the countries with the greatest species diversity of Chilocorini (Coleoptera: Coccinellidae), including nearly forty-five percent of the known genera and fourteen percent of all described species in this tribe. Recently, we discovered three species previously not recorded in China.

**New information:**

In this study, three species *Priscibrumus uropygialis* (Mulsant, 1853), *Priscibrumus disjunctus* Canepari, 1997 and *Brumus octosignatus* (Gebler, 1830) are documented for the first time in China. *Brumus octosignatus* is the first member of the genus *Brumus* Mulsant, 1850 recorded in China. Detailed descriptions, illustrations and distributions of these three species are provided. A checklist of Chinese Chilocorini is also given.

## Introduction

The members of family Coccinellidae, commonly known as colourful and shiny beetles, predators of plant pests, contain 6000 species distributed worldwide (Vandenberg 2002). The tribe Chilocorini is well-known as a primary predator of coccids. Many species of this tribe are widely used as biological control agents. In recent years, phylogenetic and evolutionary studies of Chilocorini indicated that this tribe is a monophyletic group, closely related to the tribe Coccinellini (Magro et al. 2010, Seago et al. 2011, Escalona et al. 2017, Che et al. 2017) or Plotinini (Li et al. 2020). At present, Chilocorini contains 22 genera and more than 280 species distributed worldwide (Łączyński and Tomaszewska 2012, Li et al. 2020).

China is one of the countries with the greatest species of Chilocorini (Coleoptera: Coccinellidae), including 10 genera and 39 species (Li et al. 2017a). However, in Li et al. (2017a), the genus *Brumus* Mulsant was incorrectly recorded in the Chinese checklist of Chilocorini (no literature existed to indicate that the members of this genus were distributed in China). After a phylogenetic study of the Chilocorini, the genus *Phaenochilus* Weise was synonymised with *Chilocorus* Leach (Li et al. 2020).

In this study, we report for the first time in China the genus *Brumus* with the species *Brumus octosignatus* (Gebler, 1830), as well as the species *Priscibrumus uropygialis* (Mulsant, 1853) and *Priscibrumus disjunctus* Canepari, 1997. A revised checklist of Chinese Chilocorini is also provided, containing all nine genera and 42 species.

## Materials and methods

Specimens, examined in this study, were collected in China (Tibet and Xinjiang) and deposited at the Department of Entomology, South China Agriculture University (SCAU), Guangzhou.

The newly-collected specimens of *Priscibrumus disjunctus* were identified based on the original species description (Canepari 1997). The specimens of the other two new Chinese records i.e. *Priscibrumus uropygialis* and *Brumus octosignatus*, were identified from the secondary descriptions and illustrations by Miyatake (1985) and Kovář (1997), respectively.

External morphology was observed with a dissecting stereomicroscope (SteREO Discovery V20, Zeiss). Male and female genitalia were dissected, cleared in 10% solution of sodium hydroxide (NaOH) by boiling for several minutes and examined with an Olympus BX51 microscope. Photographs of the genitalia and other morphological characters were taken with digital cameras (AxioCam HRc and Coolsnap-Procf & CRI Micro*Color), attached to microscopes using AxioVision Rel. ver. 4.8 and Image-Pro Plus ver. 6.0. Images were cleaned up and laid out in plates with Adobe Photoshop CS ver. 8.0. Terminology follows Ślipiński (2007).

Abbreviations

TL = total length: length from apical margin of clypeus to apex of elytra

TW = total width: width across both elytra at widest part

TH = body height measured across the highest point of the elytra

HW = head width in a frontal view

PL = pronotal length: from middle of anterior margin to base of pronotum

PW = pronotal width at widest part

EL = elytral length: from apex to base including scutellum

EW = elytral width, equal to TW

## Taxon treatments

### Priscibrumus

Kovář, 1997

878E2E1D-DEEB-5C59-8608-647643362168

Priscibrumus Kovář, 1997 in Kovář 1997: 114
Priscibrumus Exochomus puniceipennis Semenov, 1900

#### Diagnosis

*Priscibrumus* can be distinguished from other genera of the tribe Chilocorini by the following combination of characters: body densely covered with short, greyish pubescence; antenna composed of 10 antennomeres, with terminal antennomere very small and embedded in the penultimate antennomere; pronotal basal margin entirely bordered with submarginal line; base of pronotum and elytra contiguous all along their length; elytral epipleura narrow, more or less horizontal and without foveae; abdominal postcoxal lines almost complete; mid and hind tibiae with two apical spurs.

### Priscibrumus disjunctus

Canepari, 1997

DBFEBD22-DF86-5F7D-9C90-20724315E3A3

Priscibrumus disjunctus Canepari, 1997 in Canepari 1997: 45.

#### Materials

- **Type status:** Holotype. **Occurrence:** recordedBy: Wenjing Li; individualCount: 2; sex: 1 male, 1 female; lifeStage: adult; **Taxon:** scientificName: Priscibrumus disjunctus; **Location:** country: China; stateProvince: Tibet; locality: Jilong County; verbatimElevation: 2900 m; verbatimCoordinates: 28°18.05'N; 85°23.12'E; georeferenceProtocol: label; **Identification:** identifiedBy: Wenjing Li; dateIdentified: 2017; **Event:** samplingProtocol: sweeping; eventDate: 28/08/1984; **Record Level:** basisOfRecord: PreservedSpecimen

#### Description

TL: 3.75–4.10 mm, TW: 2.80–2.91 mm, TH: 1.83–2.12 mm, TL/TW: 1.31–1.34, PL/PW: 0.45–0.47, EL/EW: 1.07–1.10.

**Figure 1.**
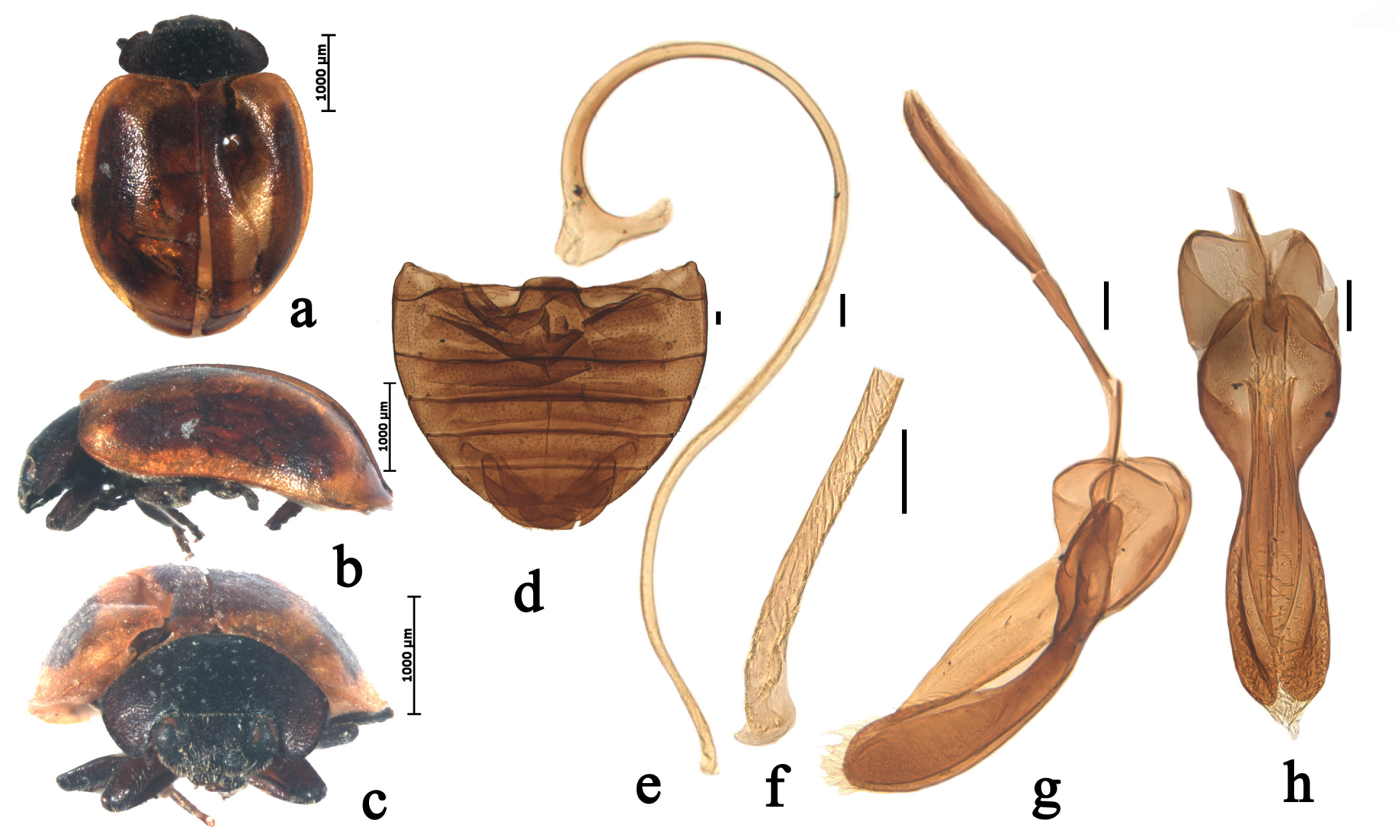
*Priscibrumus disjunctus* Canepari, 1997. **a.** dorsal view; **b.** lateral view; **c.** frontal view; **d.** abdomen; **e.** penis; **f.** apex of penis; **g.** tegmen, lateral view; **h.** tegmen, ventral view. Scale bars: 0.1 mm.

Body oval, moderately convex. Head black, densely covered with short, greyish pubescence. Mouthparts and antenna black. Pronotum black, densely covered with short, greyish pubescence. Scutellum black. Elytra reddish-brown, with two pairs of broadly black stripes: outer stripes approximately 2/5 width of elytra, 3/4 length of elytra; inner stripes situated on suture, almost as long as elytra, distinctly broadening at base and weakly broadening at apex, densely covered with extremely short, greyish pubescence (Fig. 1a–c). Underside black except elytral epipleura brownish-yellow, densely covered with short, greyish pubescence. Abdominal postcoxal lines incomplete, arcuate. Posterior margin of male abdominal ventrite 5 slightly emarginate at middle and of ventrite 6 distinctly emarginate (Fig. 1d).

Male genitalia: penis slender, penis capsule with short outer and inner arm, apex of penis truncate with membranous appendage (Fig. 1e–f). Tegmen stout, penis guide narrow at base, parallel along basal 1/3, after that, gradually broadening to basal 2/3, then narrowing to apex in ventral view; penis guide in lateral view, widest near base, gradually narrowing to apex; parameres distinctly longer than penis guide with dense short setae on the inner sides and distal end with a group of short setae in lateral view (Fig. 1g–h).

Female genitalia: coxites distinctly elongated. Spermatheca approximately C-shaped, cornu without appendage.

#### Diagnosis

This species can be distinguished from other species of *Priscibrumus* by the following combination of characters: elytra reddish-brown, with two pair of broadly black stripes, inner stripes situated on suture, almost as long as elytra; parameres distinctly longer than penis guide.

#### Distribution

Nepal (Canepari 1997) and Tibet, China (present study) (Fig. 4).

### Priscibrumus uropygialis

(Mulsant, 1853)

6F63801E-90E2-5C22-AD8C-2E493A7F22A7

Exochomus uropygialis Mulsant, 1853 in Mulsant 1853: 196.
Brumus uropygialis (Mulsant, 1853): Crotch, 1874 (see Crotch 1874: 196, Gordon 1985: 25).
Exochomus (Exochomus) uropygialis (Mulsant, 1853) Barovskij, 1922 (see Barovskij 1922: 297-8; Mader 1955: 789, 797-8; Bielawski 1979: 114; Miyatake 1985: 11).
Priscibrumus uropygialis (Mulsant, 1853): Kovář, 1997 (see Kovář 1997: 117; Canepari 1997: 45; Poorani 2002: 314).

#### Materials

- **Type status:** Other material. **Occurrence:** recordedBy: Wenjing Li; individualCount: 2; sex: 1 male, 1 female; lifeStage: adult; **Taxon:** scientificName: Priscibrumus uropygialis; **Location:** country: China; stateProvince: Tibet; locality: Mama village, Cuona County; verbatimElevation: 2800 m; verbatimCoordinates: 27°53.03'N; 91°47.34'E; georeferenceProtocol: label; **Identification:** identifiedBy: Wenjing Li; dateIdentified: 2017; **Event:** samplingProtocol: sweeping; eventDate: 25/05/2011; **Record Level:** basisOfRecord: PreservedSpecimen
- **Type status:** Other material. **Occurrence:** recordedBy: Wenjing Li; individualCount: 1; sex: 1 male; lifeStage: adult; **Taxon:** scientificName: Priscibrumus uropygialis; **Location:** country: China; stateProvince: Tibet; locality: Jilong County; verbatimElevation: 2800 m; verbatimCoordinates: 28°18.05'N; 85°23.12'E; georeferenceProtocol: label; **Identification:** identifiedBy: Wenjing Li; dateIdentified: 2017; **Event:** samplingProtocol: sweeping; eventDate: 29/05/2011; **Record Level:** basisOfRecord: PreservedSpecimen

#### Description

TL: 3.65–4.24 mm, TW: 2.72–3.31 mm, TH: 1.46–1.82 mm, TL/TW: 1.28–1.34, PL/PW: 0.46–0.51, EL/EW: 1.07–1.16.

**Figure 2.**
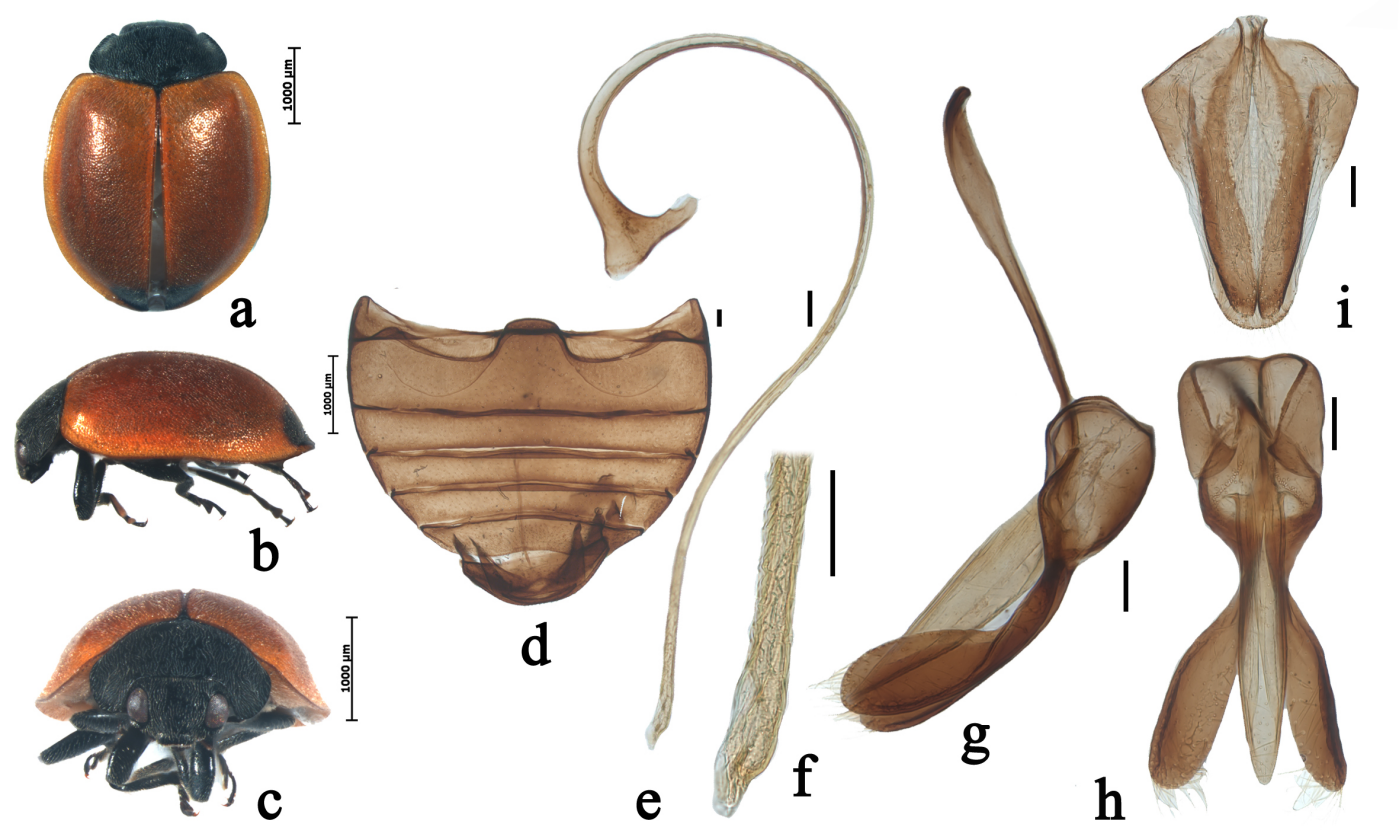
*Priscibrumus uropygialis* (Mulsant, 1853). **a.** dorsal view; **b.** lateral view; **c.** frontal view; **d.** abdomen; **e.** penis; **f.** apex of penis; **g**. tegmen, lateral view; **h.** tegmen, ventral view; **i.** ovipositor. Scale bars: 0.1 mm.

Body oval, moderately convex. Head black, densely covered with short, greyish pubescence. Mouthparts and antenna black. Pronotum black, densely covered with short, greyish pubescence. Scutellum black. Elytra reddish-brown, with a pair of black spots at elytral apex, densely covered with short, greyish pubescence (Fig. 2a–c). Underside black, except elytral epipleura brownish-yellow, densely covered with short, greyish pubescence. Abdominal postcoxal lines incomplete, arcuate. Posterior margin of male abdominal ventrite 5 slightly emarginate at middle and of ventrite 6 distinctly emarginate (Fig. 2d).

Male genitalia: penis slender, penis capsule with short outer arm and long inner arm, apex of penis truncate with membranous appendage (Fig. 2e–f). Tegmen stout, penis guide parallel along 2/3 length, then gradually narrowing to apex in ventral view; penis guide in lateral view, widest at base, parallel along 2/5 length, then gradually narrowing to apex; parameres as long as penis guide with dense long setae on the inner sides and distal end with a group of long setae in lateral view (Fig. 2g–h).

Female genitalia: coxites distinctly elongate (Fig. 2i). Spermatheca approximately C-shaped, cornu without appendage.

#### Diagnosis

This species can be easily distinguished from other species of *Priscibrumus* by the following combination of characters: elytra reddish-brown, with a pair of black spots at elytral apex; parameres as long as penis guide.

#### Distribution

Kashmir, Nepal, India, Bhutan, Pakistan (Mulsant 1850, Crotch 1874, Gordon 1987, Barovskij 1922, Bielawski 1979, Miyatake 1985, Kovář 1997, Canepari 1997, Poorani 2002, Mader 1955) and Tibet, China (present study) (Fig. 4).

### Brumus

Mulsant, 1850

49BD6CF2-085C-5C68-A9A6-62CC9C552965

Brumus Mulsant, 1850 in Mulsant 1850: 492.
Brumus Coccinella octosignata Gebler, 1830

#### Diagnosis

*Brumus* can be distinguished from other genera of the tribe Chilocorini by the following combination of characters: antenna 10-segmented, terminal antennomere very small and embedded in penultimate segment; pronotal basal margin entirely bordered with submarginal line; elytral epipleura narrow, more or less horizontal and without foveae; abdominal postcoxal lines complete; mid and hind tibiae with two apical spurs; tarsal claws without basal tooth, only slightly swollen at base.

### Brumus octosignatus

(Gebler, 1830)

E53379AA-1E43-5A00-BFDA-D1E4683C8933

Coccinella octosignata Gebler, 1830 in Gebler 1830: 225.
Coccinella deserta Motschulsky, 1840 in Motschulsky 1840: 175.
Brumus desertorum Mulsant, 1850 in Mulsant 1850: 493.
Brumus 8-signata (Gebler, 1830) Crotch, 1874 see Crotch 1874: 38.
Brumus octosignatus (Gebler, 1830) Crotch, 1874, see Crotch 1874: 195, Barovskij 1927: 196, 199; Korschefsky 1932: 199; Mader 1955: 803, 805; Bielawski 1975: 255; Bielawski 1984: 315; Kovář 1997: 36.

#### Materials

- **Type status:** Other material. **Occurrence:** recordedBy: Wenjing Li; individualCount: 2; sex: 1 male, 1 female; lifeStage: adult; **Taxon:** scientificName: *Brumus octosignatus* (Gebler, 1830); **Location:** country: China; stateProvince: Xinjiang; locality: Kezier village, Baicheng County; verbatimElevation: 850 m; verbatimCoordinates: 43°27.23'N; 82°27.40'E; georeferenceProtocol: label; **Identification:** identifiedBy: Wenjing Li; dateIdentified: 2017; **Event:** samplingProtocol: sweeping; eventDate: 31/07/1995; **Record Level:** basisOfRecord: PreservedSpecimen
- **Type status:** Other material. **Occurrence:** recordedBy: Wenjing Li; individualCount: 4; sex: 2 male, 2 female; lifeStage: adult; **Taxon:** scientificName: *Brumus octosignatus* (Gebler, 1830); **Location:** country: unknown; stateProvince: unknown; locality: unknown; verbatimElevation: unknown; verbatimCoordinates: unknown; **Identification:** identifiedBy: Wenjing Li; dateIdentified: 2017; **Record Level:** basisOfRecord: PreservedSpecimen

#### Description

TL: 3.80–4.00 mm, TW: 2.80–3.20 mm, TH: 1.87–2.07 mm, TL/TW: 1.32–1.35, PL/PW: 0.47–0.50, EL/EW: 1.05–1.10.

**Figure 3.**
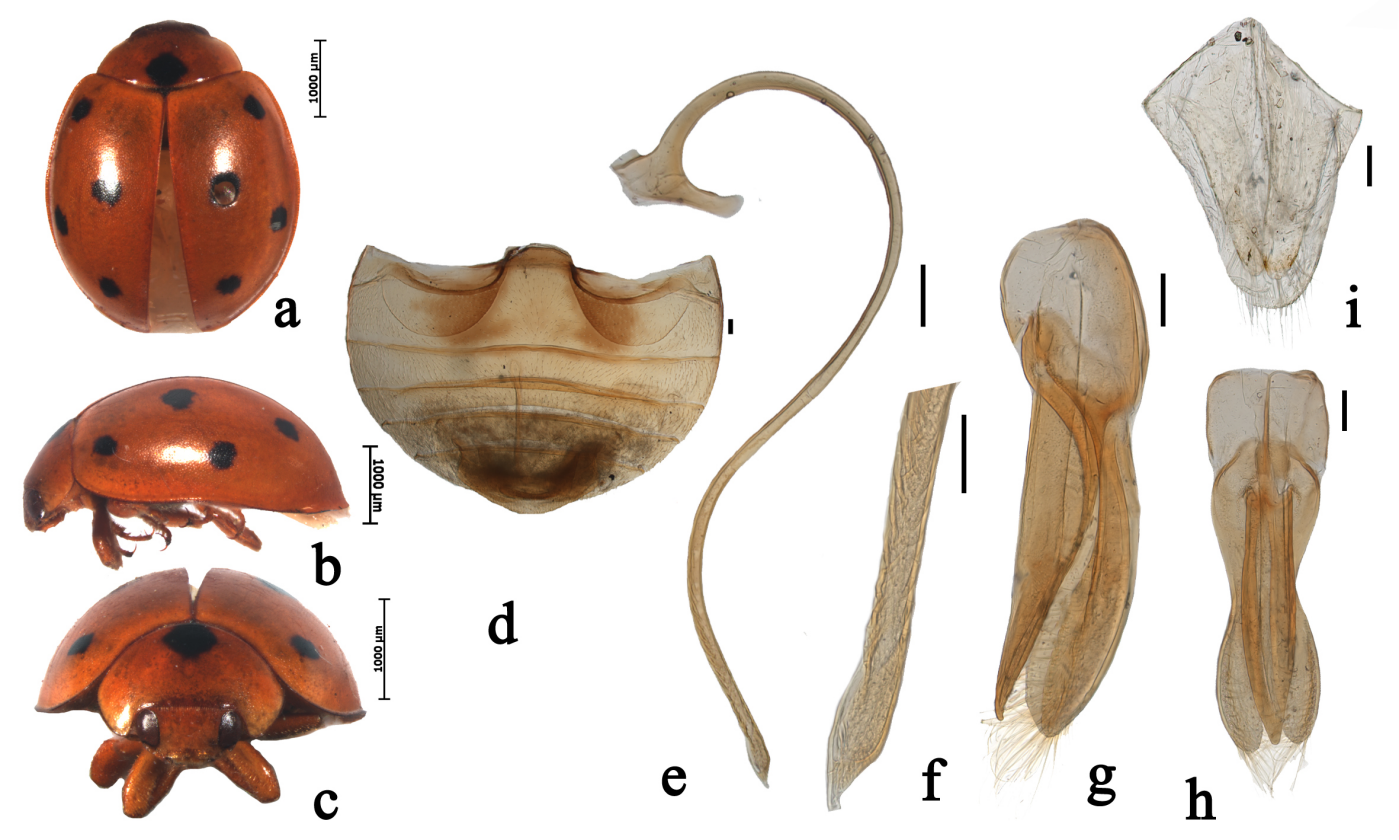
*Brumus octosignatus* (Gebler, 1830). a. dorsal view; b. lateral view; c. frontal view; d. abdomen; e. penis; f. apex of penis; g. tegmen, lateral view; h. tegmen, ventral view; i. ovipositor. Scale bars: 0.1 mm.

Body oval, moderately convex. Head, mouthparts and antenna brownish-yellow. Pronotum orange-yellow, with a black spot at centre of basal margin. Scutellum black. Elytra orange-yellow, with four pairs of black spots, the first one situated at the humeral angle; the second one situated at basal 2/5, near suture; the third one situated at basal 3/5, near outer margin; the fourth one situated basal 4/5, near suture (Fig. 3a–c). Underside orange-yellow, densely covered with short, greyish pubescence. Abdominal postcoxal lines complete and semicircular. Posterior margin of male abdominal ventrite 5 and of ventrite 6 distinctly emarginate (Fig. 3d).

Male genitalia: penis slender, penis capsule with short outer and long inner arm, apex of penis acute with membranous appendage (Fig. 3e–f). Tegmen stout, penis guide narrow at base, widest at basal 1/2, then gradually converging to blunt tip, symmetrical in ventral view; penis guide in lateral view, widest at base, parallel along basal 1/3, after that gradually converging to apex; parameres nearly as long as the penis guide with dense short setae on the inner sides and distal end with a group of short setae in lateral view (Fig. 3g–h).

Female genitalia: coxites distinctly elongate (Fig. 3i). Spermatheca approximately C-shaped, cornu without appendage.

#### Diagnosis

This species can be easily distinguished from other species of *Brumus* by the following combination of characters: pronotum with a black spot at central of basal margin; elytra orange-yellow, with four pairs of black spots; parameres nearly as long as the penis guide.

#### Distribution

**Figure 4.**
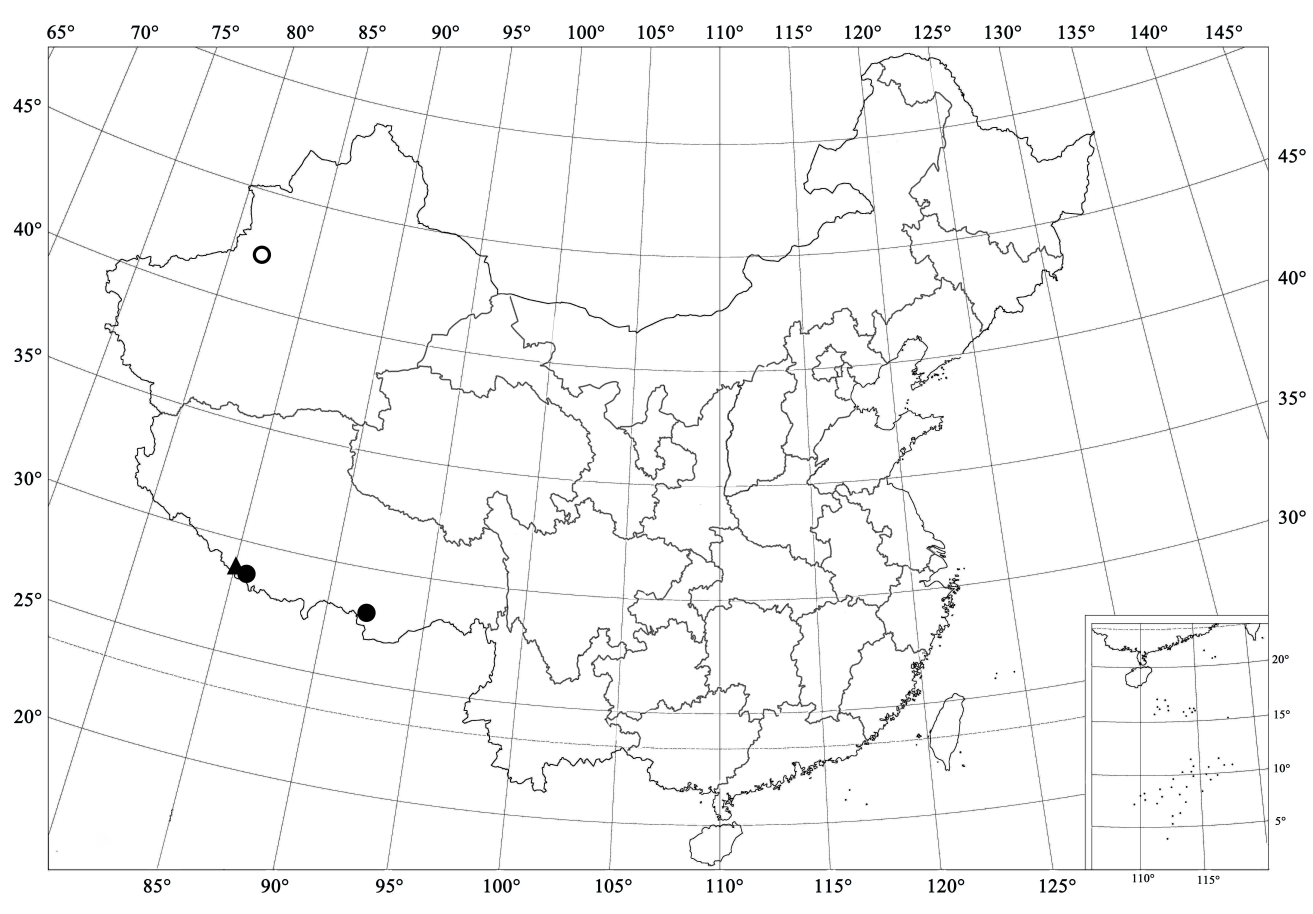
Distribution map. (▲) *Priscibrumus disjunctus* Canepari, 1997; (●) *Priscibrumus uropygialis* (Mulsant, 1853); (○) *Brumus octosignatus* (Gebler, 1830).

Azerbaijan, Armenia, France, Italy, Russia: south European Territory, Afghanistan, Iran, Iraq, Kyrgyzstan, Mongolia, Tajikistan, Turkmenistan, Turkey, Uzbekistan (Gebler 1830, Motschulsky 1840, Mulsant 1850, Crotch 1874, Barovskij 1927, Mader 1955, Bielawski 1975, Bielawski 1984, Kovář 1997) and Xinjiang, China (present study) (Fig. 4).

## Checklists

### Checklist of Chinese Chilocorini sensu Li et al. 2020

#### Brumoides

Chapin, 1965

C1D7BFF4-D441-5864-BA87-EA89FF4A197B

#### Brumoides hainanensis

Miyatake, 1970

B90B50F7-8D94-52DA-91BD-3D699476C1B6

##### Distribution

China (Miyatake 1970).

#### Brumoides lineatus

(Weise, 1885)

791DCD0B-A327-58B2-B5AF-F7FEB09F146A

##### Distribution

China, Myanmar, Thailand, Bangladesh, India, Sri Lanka, Nepal, Pakistan and Ceylon (Weise 1885, Miyatake 1970, Poorani 2002).

#### Brumoides maai

Miyatake, 1970

55161FAB-69B9-5C69-92E6-2875FA2880C5

##### Distribution

China (Miyatake 1970).

#### Brumoides ohtai

Miyatake, 1970

931B69C1-43A9-58BF-9170-6E2540BB448C

##### Distribution

China (Miyatake 1970).

#### Brumus

Mulsant, 1850

6D97AC34-4DB2-5549-A8AC-DEDA064BA76C

#### Brumus octosignatus

(Gebler, 1830)

8DCC3591-1A72-593D-BFDC-2E14429E1874

##### Distribution

China, Azerbaijan, Armenia, France, Italy, Russia: south European Territory, Afghanistan, Iran, Iraq, Kyrgyzstan, Mongolia, Tajikistan, Turkmenistan, Turkey and Uzbekistan (Gebler 1830, Motschulsky 1840, Mulsant 1850, Crotch 1874, Barovskij 1927, Mader 1955, Bielawski 1975, Bielawski 1984, Kovář 1997).

#### Chilocorus

Leach, 1815

45531EBC-C889-5E16-B124-7A819DAE4F79

#### Chilocorus albusolomus

(Li &Wang, 2017)

3AF2B3AD-6D3A-5C11-8136-600B09A769FF

##### Distribution

China (Li et al. 2017b).

#### Chilocorus alishanus

Sasaji, 1968

4905DAF8-8834-5104-B150-15FDEA4A7387

##### Distribution

China (Sasaji 1968).

#### Chilocorus bijugus

Mulsant, 1853

F09738D9-694E-5A6D-BCB3-73A84F30427D

##### Distribution

China, Japan, India, Nepal, Pakistan and Palaearctic (Mulsant 1853, Kovář 2007).

#### Chilocorus bipustulatus

(Linnaeus, 1758)

85C81AFD-2392-558A-A259-1840F0846712

##### Distribution

China, Europe, Middle East, Central Asia and North Africa (Linnaeus 1758, Li et al. 2018).

#### Chilocorus chalybeatus

Gorham, 1892

D151B203-56C4-5B92-9D2D-85DECE6E49B3

##### Distribution

China (Gorham 1892, Kovář 2007, Li et al. 2018).

#### Chilocorus chinensis

Miyatake, 1970

E87AB265-8D3D-5A20-8BB1-95BEDD3F9FF0

##### Distribution

China (Miyatake 1970, Li et al. 2018).

#### Chilocorus circumdatus

(Gyllenhal, 1808)

B65D3A0E-FA95-532C-A5B9-96252D2C718F

##### Distribution

China, Indonesia, India, Sri Lanka and introduced to Australia and America (Gyllenhal 1808, Ślipiński 2007, Kovář 2007, Li et al. 2018).

#### Chilocorus esakii

Kamiya, 1959

991E8AB0-2D2F-57CA-B2F5-A335CFB43D73

##### Distribution

China, Japan (Kamiya 1959, Li et al. 2018).

#### Chilocorus geminus

Zaslavskij, 1962

A4209690-8C5D-5A64-A0BD-E812386D1605

##### Distribution

China, Mongolia, Turkey and Central Asia (Zaslavskij 1962, Bielawski 1975, Kovář 2007, Li et al. 2018).

#### Chilocorus hauseri

Weise, 1895

5F196210-6323-5E08-AA54-1D2B3EC8D25C

##### Distribution

China, India and Myanmar (Weise 1895, Korschefsky 1932, Kovář 2007, Li et al. 2018).

#### Chilocorus hupehanus

Miyatake, 1970

68CC0E29-A578-593E-AF05-79F1D07345BB

##### Distribution

China (Miyatake 1970).

#### Chilocorus kuwanae

Silvestri, 1909

65527081-C9DB-5CD4-AF88-182BE71C2F95

##### Distribution

China, Japan, North Korea and Italy and introduced to America (Silvestri 1909, Gordon 1985, Kovář 2007, Li et al. 2018).

#### Chilocorus melas

Weise, 1898

B62642EB-2D3E-5753-8D18-49B58C1997D6

##### Distribution

China, Myanmar, Thailand, Laos, India, Nepal, Bhutan and Indonesia (Weise 1898, Miyatake 1970, Poorani 2002, Li et al. 2018).

#### Chilocorus metasternalis

(Miyatake, 1970)

894CF8A1-FF01-501F-8A29-AEF030F1DCA3

##### Distribution

China, Laos, Vietnam, Singapore and Indonesia (Miyatake 1970).

#### Chilocorus nigricaeruleus

Li & Wang, 2018

3E8BEDE9-074A-5832-B1B6-DEBE7B310BE8

##### Distribution

China (Li et al. 2018).

#### Chilocorus nigrita

(Fabricius, 1798)

EE478024-15C8-53B6-8682-8891F1CF7D91

##### Distribution

China, Myanmar, Indonesia, India, Pakistan, Sri Lanka, Bangladesh, Australian, USA, Brazil and South Africa (Fabricius 1798, Crotch 1874, Poorani 2002, Kovář 2007, Li et al. 2018).

#### Chilocorus rubidus

Hope, 1831

3D230278-A1BF-53AB-9677-1AE2D30D99D0

##### Distribution

China, Mongolia, Korea, Japan, India, Nepal, Indonesia and Siberia (Hope 1831, Kovář 2007, Li et al. 2018).

#### Chilocorus rufitarsis

Motschulsky, 1853

C587DF55-9353-54EF-8AD1-B076B8F1DCB7

##### Distribution

China and Vietnam (Motschulsky 1853, Korschefsky 1932, Kovář 2007, Li et al. 2018).

#### Chilocorus shirozui

Sasaji, 1968

F7567A00-A9F8-5784-8B73-8605A13C98AF

##### Distribution

China (Sasaji 1968, Li et al. 2018).

#### Chilocorus strenotubus

Li & Wang, 2018

FE188300-49C7-5748-918D-AABA36906CCB

##### Distribution

China (Li et al. 2018).

#### Chilocorus yunlongensis

Cao & Xiao, 1984

38FF0EAF-9949-5E22-82FB-74F3B93443BE

##### Distribution

China (Cao and Xiao 1984, Li et al. 2018).

#### Chilocorus politus

Mulsant, 1850

777DC001-0AD5-570E-88FE-5A74AA32C452

##### Distribution

China, Thailand, Laos, India, Nepal, Bhutan and Indonesia (Mulsant 1850, Poorani 2002, Kovář 2007, Li et al. 2018).

#### Chujochilus

Sasaji, 2005

83C54B32-E64B-569B-9C1A-E100C4B54F81

#### Chujochilus parisensis

Wang & Ren, 2010

D753815F-B596-59EF-BA18-D3F3EFDE8F88

##### Distribution

China (Wang and Ren 2010).

#### Chujochilus sagittatus

Wang & Ren, 2010

826D936D-5A08-5EA4-BE96-7839C7E22531

##### Distribution

China (Wang and Ren 2010).

#### Exochomus

Redtenbacher, 1843

DA672CD7-7EDD-5525-8B2A-9A972FE9FF97

#### Exochomus mongol

Barovsky, 1922

A7CDD3E8-7CF5-58CD-A735-EF070910BC7F

##### Distribution

China, Mongolia, Korea and Far East (Barovskij 1922, Kovář 1997, Kovář 2007, Li et al. 2015b).

#### Exochomus quadripustulatus

(Linnaeus, 1758)

8DA47D42-8923-5406-B997-A6986E2C5969

##### Distribution

China, Europe, Russia, Middle East and Mongolia. Introduced to USA and Australia (Linnaeus 1758, Kovář 1997, Kovář 2007, Li et al. 2015b).

#### Exochomus rubistictus

Li & Ren 2015

E206704F-7AEE-50A2-B7DE-F5A62C1DDFFC

##### Distribution

China (Li et al. 2015b).

#### Parexochomus

Barovsky, 1922

84D7CE08-742E-592B-AE56-56034C5C3ECD

#### Parexochomus nigromaculatus

(Goeze, 1777)

585E64CC-8D15-5A37-86A1-85ABC02CFE60

##### Distribution

China, Mongolia, Iran, Siberia, Arabia, Europe and Africa (Chapin 1965, Goeze 1777, Ślipiński 2007, Li et al. 2015b).

#### Parexochomus oligotrichus

Li & Ren 2015

631C7451-643A-5CBE-8045-46CB7046CBC5

##### Distribution

China (Li et al. 2015b).

#### Parexochomus semenowi

Weise, 1887

D0A9F7DF-D0B7-5D97-8F99-24CAC26050BD

##### Distribution

China and Mongolia (Weise 1887, Barovskij 1922, Kovář 2007).

#### Priscibrumus

Kovář, 1997

5018E44F-F924-5821-8237-9ACAD06A9891

#### Priscibrumus disjunctus

Canepari, 1997

B9367917-52D5-5022-A85E-AA5395813E9A

##### Distribution

China and Nepal (Canepari 1997, Poorani 2002).

#### Priscibrumus himalayensis

(Kapur, 1958)

36D37861-2E37-515F-81EB-064A0E14D041

##### Distribution

China and Nepal (Kapur 1958, Poorani 2002, Hu et al. 2013).

#### Priscibrumus uropygialis

(Mulsant, 1853)

ACCCE50E-5357-56D1-8DB5-A52FCE4B014E

##### Distribution

China; Kashmir, Nepal, India, Bhutan and Pakistan (Mulsant 1850, Crotch 1874, Gordon 1987, Barovskij 1922, Bielawski 1979, Miyatake 1985, Kovář 1997, Canepari 1997, Poorani 2002, Mader 1955).

#### Renius

Li et Wang, 2017

CA8FFA57-6831-5125-AEBD-23750A0B99B8

#### Renius cornutus

Li & Wang, 2017

7AB4E103-5BFF-5BC7-9907-56A3916AB330

##### Distribution

China and India (Li et al. 2017a, Poorani and Rojeet 2019).

#### Xanthocorus

Miyatake, 1970

EC055B36-58E0-5035-90FB-39EBB9B695A8

#### Xanthocorus mucronatus

Li & Ren, 2015

B9ED6E68-4E04-5658-A710-78CA04501A1A

##### Distribution

China (Li et al. 2015a).

#### Xanthocorus nigromarginatus

Miyatake, 1970

FE2B6E4C-4732-565A-9942-28C8359E9994

##### Distribution

China (Miyatake 1970, Li et al. 2015a).

#### Xanthocorus nigrosuturarius

Li & Ren, 2015

02083B29-0124-5BB0-905B-7DDA2BAFF015

##### Distribution

China (Li et al. 2015a).

## Discussion

The genus *Priscibrumus* was erected by Kovář (1997), when he revised *Exochomus* and *Brumus* Mulsant, 1850 from the Palearctic Region. This revision was a huge contribution to our understanding of the relationships between *Exochomus* and its closely-related genera. Until now, *Priscibrumus* only contained seven species which mainly occur in the Pamir Mountains, especially in the western part of the Himalaya Mountains (Kovář 1997, Poorani 2002). *Priscibrumus himalayensis* (Kapur) was the first member of this genus recorded in China (Tibet) by Hu et al. (2013).

*Brumus* was considered a junior synonym of *Exochomus* by Ślipiński and Giorgi (2006). Subsequently, Kovář (2007) accepted this point of view and transferred all species of *Brumus* from the Palearctic Region to *Exochomus*. The recent phylogenetic studies of Chilocorini indicated that the relationship between *Exochomus* and *Brumus* is not so close. *Exochomus* is the sister group of a large clade containing various genera, for example, *Xanthocorus*, *Priscibrumus*, *Parexochomus*, *Brumus* and *Brumoides*, while *Brumus* is closely related to *Brumoides* (Li et al. 2020). Actually, *Brumus* can be easily distinguished from *Exochomus* by the following characters: elytral epipleuron more or less horizontal; tarsal claws without basal tooth, only slightly swollen at base. In *Exochomus*, the outer part of elytral epipleuron is distinctly descending; and the tarsal claws have a large triangular tooth at the base.

## Supplementary Material

XML Treatment for Priscibrumus

XML Treatment for Priscibrumus disjunctus

XML Treatment for Priscibrumus uropygialis

XML Treatment for Brumus

XML Treatment for Brumus octosignatus

XML Treatment for Brumoides

XML Treatment for Brumoides hainanensis

XML Treatment for Brumoides lineatus

XML Treatment for Brumoides maai

XML Treatment for Brumoides ohtai

XML Treatment for Brumus

XML Treatment for Brumus octosignatus

XML Treatment for Chilocorus

XML Treatment for Chilocorus albusolomus

XML Treatment for Chilocorus alishanus

XML Treatment for Chilocorus bijugus

XML Treatment for Chilocorus bipustulatus

XML Treatment for Chilocorus chalybeatus

XML Treatment for Chilocorus chinensis

XML Treatment for Chilocorus circumdatus

XML Treatment for Chilocorus esakii

XML Treatment for Chilocorus geminus

XML Treatment for Chilocorus hauseri

XML Treatment for Chilocorus hupehanus

XML Treatment for Chilocorus kuwanae

XML Treatment for Chilocorus melas

XML Treatment for Chilocorus metasternalis

XML Treatment for Chilocorus nigricaeruleus

XML Treatment for Chilocorus nigrita

XML Treatment for Chilocorus rubidus

XML Treatment for Chilocorus rufitarsis

XML Treatment for Chilocorus shirozui

XML Treatment for Chilocorus strenotubus

XML Treatment for Chilocorus yunlongensis

XML Treatment for Chilocorus politus

XML Treatment for Chujochilus

XML Treatment for Chujochilus parisensis

XML Treatment for Chujochilus sagittatus

XML Treatment for Exochomus

XML Treatment for Exochomus mongol

XML Treatment for Exochomus quadripustulatus

XML Treatment for Exochomus rubistictus

XML Treatment for Parexochomus

XML Treatment for Parexochomus nigromaculatus

XML Treatment for Parexochomus oligotrichus

XML Treatment for Parexochomus semenowi

XML Treatment for Priscibrumus

XML Treatment for Priscibrumus disjunctus

XML Treatment for Priscibrumus himalayensis

XML Treatment for Priscibrumus uropygialis

XML Treatment for Renius

XML Treatment for Renius cornutus

XML Treatment for Xanthocorus

XML Treatment for Xanthocorus mucronatus

XML Treatment for Xanthocorus nigromarginatus

XML Treatment for Xanthocorus nigrosuturarius
